# Factors associated with smoking and transitioning to nyaope injection amongst women in the City of Tshwane Municipality: A self-report by women

**DOI:** 10.4102/hsag.v27i0.1775

**Published:** 2022-07-18

**Authors:** Moganki H. Lefoka, Robert T. Netangaheni

**Affiliations:** 1 Community Oriented Substance Use Programme, Department of Family Medicine, Faculty of Health Sciences, University of Pretoria, Pretoria, South Africa; 2 Department of Sociology, Faculty of Human Sciences, University of South Africa, Pretoria, South Africa; 3 Department of Health Studies, Faculty of Human Sciences, University of South Africa, Pretoria, South Africa

**Keywords:** nyaope, people who use drugs, women who inject drugs, Needle and Syringe Exchange Programme, substance use disorder

## Abstract

**Background:**

Substance use disorder has emerged as a key health and social challenge in South Africa (SA). It is projected that about 15% of South African youth, especially young women are prone to engage in drug use and the prospects of coming into contact with nyaope, a highly addictive drug, are higher. Nyaope is mainly smoked, but the prevalence of injecting it is increasing in most regions.

**Aim:**

This study aimed to explore and describe the perception of women, who use nyaope, about the factors contributing to nyaope smoking and transitioning to injecting nyaope amongst women in the City of Tshwane Municipality (CoT), Gauteng.

**Setting:**

The research was conducted within Community Oriented Substance Use Programme sites across the CoT Municipality.

**Methods:**

Qualitative research methods were utilised to explore and describe the perceptions of the participants on factors contributing to the use of nyaope amongst women residing in the CoT. Data were collected through face-to-face interviews and thematically analysed.

**Results:**

Intimate partner influence (IPI), peer pressure, being misled by friends, weight loss, lack of effective coping mechanisms and counteracting other drugs contributed to women smoking nyaope. Additionally, a need for an intense high, IPI, influence by the social network, curiosity and cost-effectiveness contributed to women transitioning from smoking to injecting nyaope.

**Conclusion:**

The study has established factors contributing to smoking and transition to injecting nyaope as viewed by women residing in the CoT.

**Contribution:**

This research affirms that women are influenced by different factors to use and transition to injecting Nyaope.

## Introduction

Substance use disorder (SUD) has emerged as a major public health and social issue in South Africa (SA), with children as young as 8 years old reporting using nyaope (Masombuka 2013; Masombuka & Qalinge 2020; Mokwena & Morojele 2014). The SUD has presented itself as an overwhelming challenge both globally and in SA; it is a complex challenge that undermines the social fabric of humanity and has a negative impact on the lives of both users and non-users alike (Charlton, Negota & Mistry 2019; Fernandes & Mokoena 2020; Mokwena & Morojele 2014). Social pressure, peer pressure, a dysfunctional family, genetic factors, emotional problems, mental health problems, loss of respect, trauma experience, loss of livelihood and prior substance exposure have all been identified as contributing factors to SUD (Mokwena, Shandukani & Fernandes 2021; Pourallahvirdi et al. 2016).

It is projected that about 15% of South African youth, including young women, are prone to engage in drug use, and the prospects of coming into contact with nyaope are higher (Fernandes & Mokwena 2016). The use of nyaope has a significant impact on one’s health, society and finances (Tetarwal et al. 2019); nyaope also causes irreversible mental and social harm to the individual (Fernandes & Mokwena 2020).

According to statistics, drug consumption in SA is nearly twice that of the rest of the world and it is the highest in Africa (Charlton et al. 2019). A steady increase in the consumption of several drugs has caught SA off guard, with nyaope leading the way (Bala & Kang’ethe 2021). Despite the rise in the prevalence of nyaope through smoking and injections in SA, the South African government has not fully embraced the harm reduction approach as one of the frameworks for addressing the public health concerns that are unavoidably associated with the use of nyaope (Lefoka & Netangaheni 2021). Almost all of SA’s SUD treatment funding goes to abstinence-based programmes (Scheibe et al. 2017). There is only one harm reduction programme financed by the South African government. The Community Oriented Substance Use Programme (COSUP), run by the University of Pretoria’s Department of Family Medicine and sponsored by the City of Tshwane Municipality (CoT), is SA’s first publicly funded community-based harm reduction response to the use of unregulated drugs (Scheibe et al. 2020a).

Nyaope is one of the drugs that is of concern in such programmes as COSUP. Nyaope is a trendy new drug popular in SA’s townships. Nyaope’s full composition is unknown, despite the fact that most researchers agree that heroin is the primary ingredient, with rumours of antiretroviral medication being documented (Lefoka & Netanghaheni 2021). It is reported that when users attempt to stop using nyaope on their own, they experience severe withdrawal symptoms such as severe unbearable abdominal cramps (Khine & Mokwena 2016; Meel & Essop 2018; Mthembi, Mwenesongole & Cole 2018).

Although nyaope was rolled and smoked with cannabis at first, there is a growing trend of dissolving nyaope in water and injecting it directly into the user’s veins (Fernandes & Mokwena 2020). The process of transitioning from smoking to injecting is viewed as a process of coping with a variety of resource constraints and a process of curiosity and pleasure seeking (Guise et al. 2015). Nyaope injection is presently an increasing trend in most regions of SA (Weich et al. 2017). Baluku and Wamala (2019) found that women are easily influenced by their male peers and clients to transition to injecting, especially if they work in the sex industry. Women who use drugs are more vulnerable than men to HIV and other blood-borne infections; not only for biological reasons but also because of gender power disparities. For example, being incapable of discussing condom use, being injected after an intimate male partner has injected himself with the same needle and being involved in sex work (UNODC 2018). The injecting practice, as compared with smoking, comes with countless risk factors for women, including the risk associated with injecting intimate partners and sharing unsterile injections (Tuchman 2015). Whilst all methods of taking nyaope have risks, injecting is the most dangerous because it is connected to a variety of health and social problems, including HIV, hepatitis C, skin and vein damage and arrest and imprisonment (Guise et al. 2015). The consequences of drug use are more severe in women than in men, and they are linked to issues such as financial hardship, increased drug dependence, health risks and involvement in HIV-related high-risk behaviours (Jamshidimanesh et al. 2016). Women with limited financial resources or career options are more likely to engage in sex work, increasing their HIV risk and adding to the stigma associated with sex work (Larney et al. 2015).

In townships, people who use nyaope are often labelled as ‘nyaopers’: a derogative name, meaning nyaope user and it perpetuates stigmatisation of the users (Mokwena 2016). The stigmatisation contributes to communities distancing themselves from individuals who use drugs and being afraid of those with drug use difficulties, viewing them as dangerous (Paquette, Syveertsen & Pollini 2018). Those who use nyaope are the most stigmatised amongst key populations in SA, limiting their access to healthcare and protection (Bala & Kang’ethe 2021). This is further fuelled by the traditional stereotypes that hold women to different expectations and roles as mothers, partners and caretakers, women who inject drugs are stigmatised more than men (El-Bassel & Strathdee 2016; Khuat et al. 2015).

In women, the effects of drug use are more severe than in men. They are linked to financial hardship, increased drug dependence, health risks and participation in HIV-related high-risk behaviours (Jamshidimanesh et al. 2016). Furthermore, according to a UNODC study conducted in SA in 2018, women who use drugs (WWUDs) are subjected to human rights violations on all levels (UNODC 2019).

Women are more likely than men to begin injecting drugs with an intimate partner and to have that partner inject them (El-Bassel & Strathdee 2015; Global Coalition on Females and AIDS [GCWA] 2011). Women who are in an intimate relationship with a drug user are significantly associated with the initiation and continuation of drug use in women (UNODC 2018). They are also more likely to request injections from their male partners, which contributes to their increased vulnerability (GCWA 2011; UNODC 2018).

Furthermore, WWUD have a high rate of intimate partner violence (IPV) (Larney et al. 2015). Intimate partner violence is more common amongst WWUD than amongst women in general (Stoicescu et al. 2018). According to some studies, WWUD experience 3–5 times more physical and sexual IPV than women in the general population (Pinkham, Stoicescu & Myers 2012).

People who inject drugs (PWIDs) are more likely to experience mental health issues such as depression, psychosis, anxiety and personality disorders. It is well understood that mental health issues lead to drug use (UNODC 2015). Several studies have found a higher prevalence of SUDs in people with mental illnesses (range of 21.3% – 55.6%) than in the general population (3% – 4%) (Tindimwebwa, Ajayi & Adeniyi 2021). Women are more likely than men to attribute their substance use to trauma or stressors such as relationship issues, environmental stress or family issues (UNODC 2018). Women’s drug use can be exacerbated by mental health issues, which limits their ability to recognise and navigate potentially dangerous situations (Stoicescu et al. 2018). Trauma can cause mental health problems and increase the likelihood of using drugs (Arpa 2017).

Substance use disorder is a multifaceted problem caused by a variety of factors. It requires a varied approach; it presents unique challenges for police and other law enforcement agencies; SUD should be addressed to assist people who use drugs (PWUDs) rather than arresting and prosecuting them, thus requiring an integrative approach to address nyaope challenges in communities (Dintwe 2017). Users’ challenges, coping resources, experience, risks, needs and hopes cannot be addressed without involving them in the process (Lefoka & Netangaheni 2021).

Despite an alarming increase in substance abuse amongst women around the world, the issue is largely ignored and neglected (Bala & Kang’ethe 2021). Such ignorance is puzzling because WWUDs face greater stigma than their male counterparts (Rahman et al. 2015). Women who use drugs are rarely the subject of research, their reasons for use are unclear and poorly understood (El-Bassel & Strathdee 2016; UNAIDS 2014). Policymakers would appreciate the complexity of SUD if the researchers and practitioners had a clear understanding of the reasons why women use drugs, rather than assuming that drug use is merely a matter of individual choice that abstinence and prohibition can solve, rather than a symptom of social problems (Mburu et al. 2018).

## Aim

This study aimed to explore and describe the women’s perception of factors contributing to nyaope smoking and transitioning to injecting nyaope amongst women in the CoT Municipality, Gauteng.

## Research methods and design

### Study design

Qualitative methods were utilised to explore and describe the women’s perceptions of factors contributing to nyaope smoking and transitioning to injecting nyaope amongst women in the CoT Municipality, Gauteng. Qualitative research is commonly used when the research aim is to obtain an understanding of people’s perspectives about the world. Furthermore, it is used to answer questions about people’s experiences, perspectives and meanings from their own understanding (Creswell & Creswell 2018). Qualitative research method was the most appropriate research paradigm because it allowed the researchers to focus on describing and understanding factors that contribute to nyaope smoking and transition to injecting nyaope amongst women residing in the CoT, rather than explaining factors that contribute to the use of nyaope amongst the CoT women, as the women themselves experienced.

### Research question

The following research question guided the study: What are the women’s perceptions of factors contributing to nyaope smoking and transitioning to injecting nyaope amongst women in the CoT Municipality, Gauteng?

### Setting

The study took place in COSUP sites across the CoT Municipality. Data were gathered at eight of 17 COSUP sites across the CoT Municipality. Prior to visiting further COSUP sites for data collection, the researchers reached data saturation. Community Oriented Substance Use Programme was established on a system thinking, harm reduction approach to public health and clinical care. It seeks to provide evidence-based, substance use services integrated into community oriented primary care (Lefoka 2019; Scheibe et al. 2020a).

### Study population and sampling strategy

The population in this study were women above the age of 18 years who reside in the CoT Municipality with a history of injecting nyaope for more than 6 months. The participants were accessing harm reduction services at COSUP sites across the CoT. The purposive-sampling method was used to recruit participants from COSUP needle and syringe exchange programme (NSP) and opioids substitute therapy (OST) social work caseload. All women accessing services at COSUP with a history of injecting nyaope who meet inclusion criteria were invited through the assistance of the site social workers to participate in the study. The researcher sampled 24 participants from the population with the help of site social workers. Fifteen women who were currently injecting nyaope and nine women with a history of injecting nyaope were interviewed. Inclusion and exclusion criteria were followed during the sampling process (Table 1 and Table 2). The researcher was more interested in interviewing women who had a rich life experience of the phenomenon being studied, in this case, experience of using nyaope. Injecting nyaope for 6 months was deemed sufficient to determine whether or not a person has extensive experience, those with less than 6 months, were not considered.

**TABLE 1:** Inclusion criteria.

Women who recovered from injecting nyaope	Women who currently inject nyaope
The participant must be a female with 6 months or more of injecting nyaope before stopping	The participants must be female with 6 months or more history of injecting nyaope
The participant must live in the City of Tshwane Municipality	The participant must live in the City of Tshwane Municipality
The participant must be 18 years old and above	The participant must be 18 years old and above

*Source*: Lefoka, M.H., 2019, ‘Exploring the experiences of women injecting nyaope residing in the City of Tshwane Municipality’, Gauteng’, Dissertation for Masters of Social Behavioural Studies in HIV/AIDS, University of South Africa.

**TABLE 2:** Exclusion criteria.

Women who recovered from injecting nyaope	Women who currently inject nyaope
Participants who are intoxicated	Participants who are intoxicated
Participants who are not linked to an organisation	Participants who are not linked to an organisation
Participants who required financial incentives to participate in the study	Participants who required financial incentives to participate in the study

*Source:* Lefoka, M.H., 2019, ‘Exploring the experiences of women injecting nyaope residing in the City of Tshwane Municipality’, Gauteng’, Dissertation for Masters of Social Behavioural Studies in HIV/AIDS, University of South Africa.

### Data collection

Permission to conduct the study was granted by COSUP management before data were collected. The researcher liaised with the site social workers for data collection. In preparation for data collection, the social workers handed participants’ consent forms and assisted those who could not understand English with understanding the content of the forms. The consent forms were written in simple English, free of jargon and legalese for participants to comprehend the meaning before participating. The researcher also checked with the participants if they understood the purpose of the research before collecting data. The risks, benefits and rights were discussed in the presence of a social worker before they consented to participate in the study. All participants were allowed an opportunity to accept or decline to participate in the research study and no incentives were offered in any form. One participant did not want to participate and the researcher respected the decision.

Data were collected using semi-structured interviews with 24 participants (see interview schedule). All the interviews were audio-recorded and the interviews were kept safe to protect the information and maintain confidentiality. All the audio recordings and transcript were kept in an encrypted file in a password protected computer. The researcher initially envisaged interviewing 30 participants, 15 recovered injectors and 15 current injectors to enable the researcher to collect rich data from multiple sources across the CoT. However, 15 current injectors and 9 recovered injectors were interviewed. The researcher did not interview 15 recovered injectors as envisaged because of data saturation. The researcher stopped the interview after reaching data saturation. Saturation was reached after the 19th interview, but the researcher continued until the 24th interview. The researcher wanted to make sure that the interview process was not stopped prematurely. As a result, five more interviews were conducted after data saturation.

The researcher encountered some challenges during data collection. For example, one participant requested money to participate in the study. She indicated that she did not have money for drugs and must go and look for money and participating in the study would waste her time. The researcher did not give her money and she left. In addition, two potential participants were intoxicated during data collection; as a result, they were not considered for inclusion.

### Semi-structured interview guide

- Demographic questions
  1. Gender……………………………………………
  2. Age………………………………………………
  3. Duration of injecting nyaope……………………
  4. Duration without injecting nyaope………………….
- Research questions
  1. How did you start to use nyaope?
  2. What influenced you to inject nyaope?

### Data analysis

Data were analysed using six phases of thematic analysis as identified by Braun and Clarke (2006).

**Phase 1:** The researchers acquainted themselves with the data.

The audio recordings of the 24 interviews were verbatim transcribed by the researchers. The researchers were able to familiarise themselves with the content of the transcripts through the transcription process.

**Phase 2:** The researcher generated initial codes.

The researchers went through the transcripts one by one, critically identifying initial codes, allowing them to code a large number of codes that could form different themes.

**Phase 3:** The researcher searched for themes.

The researchers began by grouping together codes that were related to one another into possible themes. Finally, the researchers used a tabular form to neatly organise the codes and themes.

**Phase 4:** The researcher reviewed themes.

All of the themes were reviewed by the researchers. Sub-themes were created to accommodate themes that are complementary.

**Phase 5:** The researcher defined and named the themes.

This phase began when the researchers were satisfied with the themes and sub-themes identified. It was determined what each theme was about and what type of data each theme captured.

**Phase 6:** The researcher produced the report.

The researchers began writing up the data’s thematic analysis. The researchers worked hard to provide a succinct, coherent, logical, non-repetitive and interesting account of the factors that contribute to nyaope smoking and transitioning to injecting nyaope amongst women.

### Ethical considerations

This article is part of a MA dissertation entitled: ‘Exploring the experiences of women injecting nyaope residing in the City of Tshwane Municipality, Gauteng’. It was approved by the Research Ethics Committee of the College of Human Science, University of South Africa, with reference number 2019-CHS-0246. Permission to collect data within COSUP sites was granted by the University of Pretoria, Department of Family Medicine. All participants provided informed consent to participate in the study. The participation was voluntary, and the researcher adhered to social science ethics throughout the study.

### Measures to ensure trustworthiness

The standard of good qualitative research is based on trustworthiness. Trustworthiness is based on four constructs: credibility, transferability, dependability and conformability (Kumar 2014).

#### Credibility

Credibility confirms that the results of qualitative research are credible or authentic from the participants’ perspective in the study (Kumar 2014). The findings must reflect the views of the participants as they were shared by the participants. The researcher enhanced credibility by:

- Building rapport with the participants: Pre-counselling was conducted with research participants before data collection to build rapport.
- Transcript verification: Four transcripts were taken to the participants to read through and confirm the information as the interviews were conducted in Sepedi (Sepitori) then translated to English. Transcripts were taken back so that the participants could establish if the interview transcript did not lose meaning during the translation process.
- Two participants who were under the influence of the substance(s) at the time of the interview were not considered.

#### Transferability

Transferability is described as a level to which the results of qualitative research can be generalised or transferred to other contexts of setting (Kumar 2014). The researcher has interviewed multiple participants from different areas within the CoT Municipality. The researcher has audiotaped the interview, observed and explored nonverbal messages during the interview.

#### Conformability

Conformability is described as the level to which the results can be confirmed or corroborated by other researchers (Kumar 2014). De Vos et al. (2011) reported that conformability captures the traditional concept of objectivity. Conformability was captured by audio recording of the participants, following up on questions, probing and not assuming what the researcher did not understand.

#### Dependability

Dependability is described as whether the same results will be achieved if the same phenomenon is observed twice. Qualitative research promotes flexibility and freedom (Kumar 2014). De Vos et al. (2011) stated that the researcher should ask whether the research process is logical, well documented and audited to improve dependability. As a result of the high cost, the researcher could not appoint an independent co-coder; instead, the supervisor, as an experienced qualitative and mix-method researcher, assisted with co-coding to ensure dependability.

### Biographic characteristics of the patients

Table 3 contains the biographical characteristics of the participants.

**TABLE 3:** Biographical characteristics of the participants.

Participant	Employment status	Age	Duration of injecting nyaope (years)	Duration without drugs
1	Unemployed	23	7	Currently injecting
2	Unemployed	32	4	Currently injecting
3	Unemployed	34	5	Currently injecting
4	Unemployed	22	4	Currently injecting
5	Unemployed	31	5	Currently injecting
6	Unemployed	27	6	Currently injecting
7	Unemployed	24	8	Currently injecting
8	Unemployed	35	13	Currently injecting
9	Unemployed	31	11	Currently injecting
10	Unemployed	32	12	Currently injecting
11	Unemployed	35	9	Currently injecting
12	Unemployed	32	4	Currently injecting
13	Unemployed	28	1	Currently injecting
14	Unemployed	23	4	Currently injecting
15	Unemployed	27	9	Currently injecting
16	Unemployed	31	6	5 months
17	Unemployed	27	10	1 year 8 months
18	Unemployed	32	6	8 months
19	Unemployed	30	9	4 months
20	Unemployed	35	16	5 months
21	Unemployed	26	6	6 years
22	Unemployed	32	15	9 months
23	Unemployed	31	12	5 months
24	Unemployed	29	9	11 months

*Source:* Lefoka, M.H., 2019, ‘Exploring the experiences of women injecting nyaope residing in the City of Tshwane Municipality’, Gauteng’, Dissertation for Masters of Social Behavioural Studies in HIV/AIDS, University of South Africa.

## Results

Data were collected from a sample of twenty-four women with a history of injecting nyaope. Two main themes and eleven sub-themes emerged from the data analysis (see Table 4).

**TABLE 4:** Themes and sub-themes.

Themes	Sub-themes
Factors contributing to the smoking of nyaope	Intimate partner influence Peer pressure Being misled by friends Using nyaope for weight loss Lack of effective coping mechanisms Counteracting other drugs
Factors contributing to transition from smoking to injecting nyaope	Need for an intense high Intimate partner influence Influence by the social network Curiosity Cost-effectiveness

### Theme 1: Factors contributing to the smoking of nyaope

Factors contributing to the smoking of nyaope were the first theme identified in this study. Sub-themes are discussed in the following subsection.

#### Sub-theme 1.1: Intimate partner influence

Participants report that they started using nyaope with their intimate partner. One participant further alluded that the desire to spend time with her intimate partner contributed to her decision to use nyaope. She was of the view that, if she uses Nyaope, her intimate partner will spend time with her. This is supported by the following narratives:

‘I was trying to please my boyfriend. I loved him, and I wanted him to spend time with him’ [*giggling*]. (Participant 19, Female, 30 years old)

‘I was in a relationship with someone using nyaope. I was using Cat at that moment and from Kat [*methcathinone*] he introduced me to Nyaope.’ (Participant 13, Female, 28 years old)

‘I first started using nyaope with my boyfriend.’ (Participant 12, Female, 32 years old)

#### Sub-theme 1.2: Peer pressure

Peer pressure and a sense of belonging contribute to nyaope use amongst women. Friends play a crucial role in introducing a woman to substance use. Some participants reported that they started to use nyaope with friends. Smoking dagga with friends cultivates a ground to initiate nyaope use; it makes it easier to try new drugs, including nyaope. One of the participants further reported that a desire to feel she belongs or fit in her social group influenced her decision to use nyaope as she did not want to be considered ‘uncool’, the need for acceptance got the participants in drugs. Participants reported that:

‘Truly speaking, I was involved [*in drug use*] because of peer pressure, having friends at school who are smoking and feeling like if you do not do what they do, you are not cool, that is how I got hooked.’ (Participant 10, Female, 32 years old)

‘I was with my friends, we were smoking dagga, and they taught me to smoke nyaope.’ (Participant 24, Female, 29 years old)

‘I was with my friends, we began by smoking dagga then this other day our friend came with nyaope for us to try out. That is how I was introduced to nyaope.’ (Participant 23, Female, 31 years old)

#### Sub-theme 1.3: Being misled by friends

Some participants have indicated that friends misled them into smoking nyaope. They were not aware that they were smoking nyaope when they started to smoke nyaope. They assumed they were smoking dagga only to discover their friends mixed dagga with nyaope. Participants reported that:

‘This friend was smoking and doing a lot of things. They would give me to smoke, indicating that it is only dagga. I was surprised this other morning when I woke up, I started to feel stomach pains, but I did not understand until she came and told me to give her money so that she can buy [*Nyaope*]. After smoking, the pains disappears.’ (Participant 9, Female, 31 years old)

‘I lived with this guy (who is my neighbour) in a two-room [*house*]. I had borrowed [*rented*] him the other room so that when I am not there, he can guard for me. That guy was smoking nyaope. One day I told the guy to prepare the dagga because I was still busy cooking; I will come and smoke. He then prepared the dagga. I was not paying attention when he was doing it as I was busy. Whilst he was preparing, he added nyaope. I am used to preparing mine with a paper from yellow pages book; that guy was preparing it with rizla. I went to his room to smoke; I felt that this dagga is different from the one that I am smoking [*on regular occasions*] then I asked him why is dagga it tasted different and it made me dizzy. He said this is the one that is good. Then I started to like that one that he prepared instead of the one I prepare myself because but the other one I feel the high same time [*compared with the other one which takes time*]. By then I was not aware that it is that thing [*Nyaope*].’ (Participant 3, Female, 34 years old)

#### Sub-theme 1.4: Using nyaope for weight loss

Weight loss has been identified as a factor that influenced participants to start using nyaope. Their initial idea was to lose weight, but they got dependent on nyaope, and continued using. Some participants exhibited poor decision-making processes in their pursuit to lose weight. Participants reported that:

‘For me to start using nyaope was because I wanted to lose weight, but I got addicted.’ (Participant 18, Female, 32 years old)

‘I started smoking nyaope because I want to slim, to lose weight.’ (Participant 16, Female, 31 years old)

#### Sub-theme 1.5: Lack of effective coping mechanism

Most of the participants reported that they started using nyaope because of issues they were facing in life. Participants started using nyaope as coping strategies to deal with the stressful events they were experiencing in their lives. One participant spoke of how she witnessed the murder of her father, whilst another talked of rape by her adoptive father and rejection. The incidents influenced them to use nyaope to cope with what they were experiencing. Participants reported:

‘It was when my brother killed my father, it [*the murder*] has affected me a lot. When I tried to reflect on what happened, it was just too much.’ [*teary eyes*] (Participant 20, Female, 35 years old)

‘I started to smoke nyaope because of the things that happened in my life. I was adopted, and [*through that*] adoption, my father turned me into his sex slave. When I spoke with my adoptive mother, she told me that I was lying about it. That is when I was told how they did a favour for me, how they picked me from gutters, how I should be grateful. So they ended up kicking me out, and I stayed on the streets.’ (Participant 15, Female, 27 years old)

‘My brother had just passed away; I was stressed and I could not sleep. I had a friend who was smoking nyaope; she told me that if I can smoke this (nyaope), I will be able to sleep.’ (Participant 21, Female, 26 years old)

#### Sub-theme 1.6: Counteracting other drugs

The participants started using nyaope to counteract other drugs. Participants were using cocaine, which is a stimulant. They introduced nyaope to mitigate lack of sleep as a result of cocaine use. It was never their intention to be regular users, but because of the addictiveness of nyaope, the participants were later dependent on nyaope. Participants reported that:

‘I was using uppers; I was using cocaine. You know, with cocaine, it is like when you want to sleep it is a problem; so I knew that this thing [*nyaope*] will help me to sleep.’ (Participant 4, Female, 22 years old)

‘I met some people who were smoking [*cocaine*] but they told me about the downer. The downer is heroin, it pins you down when you’re too up [*too high*].’ (Participant 2, Female, 32 years old)

### Theme 2: Factors contributing to transition from smoking to injecting nyaope

Factors contributing to the transition from smoking to injecting nyaope were the second theme identified in this study. Sub-themes are discussed the following subsections.

#### Sub-theme 2.1: Need for an intense high

The need to experience an intense high lasting longer has influenced participants to transition from smoking nyaope to injecting it. The transition from smoking dagga to injecting initiation was based on the need to increase the drug’s effect. The majority of the participants indicated that when nyaope is injected, it gives a high faster than smoking nyaope. The need for intense high contributed to the transition. Participants reported that:

‘I was smoking, but [*after*] realising that I do not feel that kick that I want, so I went for an injection.’ (Participant 8, Female, 35 years old)

‘When I started injecting, smoking dagga was not giving me a desired high. I did not feel it after smoking; so a friend told me about the injecting practice. I was told if I inject it remains longer in my body and I will achieve the desired high.’ (Participant 10, Female, 32 years old)

‘Started injecting because smoking it did not do anything anymore [*did not give me a high*].’ (Participant 4, Female, 22 years old)

‘I felt that smoking dagga was not strong like it used to be and when I injected, it stayed longer in the body but zolo [*dagga*] did not.’ (Participant 15, Female, 27 years old)

#### Sub-theme 2.2: Intimate partner influence

The participants were influenced by their intimate partners to transition from smoking nyaope to injecting. The influence of intimate partners on initiating injecting nyaope should be acknowledged. Such an influence increases the risk of female nyaope injectors contracting HIV and other blood-borne infections. Participants reported that:

‘Because the father of my child, the person I was dating was smoking zol and ended up injecting, I also ended up injecting.’ (Participant 17, Female, 27 years old)

‘My boyfriend influenced me [*to inject*], sometimes when we do not have money, he was injecting and I was not [*injecting*]. This other time he said let me allow you to try. Mostly he would say that but I would refuse [*to inject*] but on that day because we only had one bag, he mixed then that is how I started.’ (Participant 1, Female, 23 years old)

#### Sub-theme 2.3 Influence by social network

Participants were introduced to injecting practice by people in their social circle who were already injecting. The friends enticed participants by informing them about the benefits of injecting nyaope, but did not inform participants about the risks of engaging in injecting practice. The participants reported that:

‘I met this other guy who was injecting. He taught to me inject. He just said it is nice, you will enjoy. He even told me that it stays longer in the blood than zolo [*dagga*]. I just said that spike me and then it continued and it was like that since.’ (Participant 11, Female, 35 years old)

‘It was a friend I got to know, I moved to Cape Town and met this friend. He was a white guy; he was injecting and I was smoking so he told me I am going to feel it more. I can feel the nyaope more if I inject because I was complaining to get more high. He injected me, he taught me how to inject.’ (Participant 2, Female, 32 years old)

#### Sub-theme 2.4: Curiosity

Women who inject nyaope are not on all occasions inactive in transitioning from smoking to injecting drugs. Often, they have an active role in transitioning. Some participants reported that the transition was their own idea. For the participants, as highlighted, curiosity was a contributing factor, which influenced them to inject nyaope. The participants reported that:

‘I saw people injecting so I became curious.’ (Participant 23, Female, 31 years old)

‘I was smoking at Soshanguve and some boys were injecting with one girl but they got the intense high more than us who are smoking with cocktail, then I got curious.’ (Participant 16, Female, 31 years old)

‘I was dating my boyfriend who was also a smoker. It happened that this other day we found new needles and we used to see people injecting and we were smoking cocktail. He said to me let us sell these needles then I said no! Let us not sell them let us try to inject and feel what they feel only for today.’ (Participant 7, Female, 24 years old)

#### Sub-theme 2.5 Cost effective

Nyaope comes at a high price. The majority of participants indicated that injecting nyaope reduces the cost as one achieves an intense high and remains in the body for longer. Injecting nyaope reduces the cost in the sense that the user uses less to get an intense high. The participants reported that:

‘The problem of cocktail [*mixing with Nyaope with dagga*] is that I would smoke the whole day because after an hour I withdraw, then I have to smoke, and it becomes costly. But with injecting you can smoke two packets; you can stay the whole day with the desired high until late at night.’ (Participant 16, Female, 31 years old)

‘It was going to be too much costly for me, the more I buy the more money I need to have. It is better for me to inject so that I can have the satisfying high.’ (Participant 24, Female, 29 years old)

## Discussion

The findings of this study affirm that there are factors that influence women’s decisions to smoke nyaope and to transition to injecting nyaope. It also shows that nyaope is used by women, and researchers should consider studying WWUDs in order to gain a better understanding of their experiences. Data from the Western Cape Province of SA show that amongst women seeking treatment for substance abuse, the proportion of heroin-related admissions increased significantly from 4.8% in 2000 to 5.6% in 2013 (Morgan, Daniels & Subramaney 2020). With the increase in women who smoke drugs and women who inject drugs, further delay can increase poor policymaking and irrelevant provisions of SUD programmes.

Furthermore, the findings demonstrate that an intimate partner influence plays an important role in influencing women to smoke nyaope and further, transition to injecting. Women are more likely to start using drugs as a result of family issues and the influence of their intimate relationships, whereas men are more likely to be introduced to drug use through their peer and friendship networks (Zolala et al. 2016). Being in an intimate relationship with a drug-using partner has been shown to have a significant impact on a woman’s decision to begin and continue drug use (GCWA 2011). Although the intimate relationship between the women and injecting partner is complex (Azim, Bontell & Strathdee 2015), gender inequality and power imbalances characterise the relationship, making women particularly vulnerable (Morrisa et al. 2018). Societal customs that suppress women and build imbalanced power relationships further strengthen injection sharing and drug-related norms in intimate partnerships (Marotta et al. 2018).

The findings of the study attest that some women started and consequently became addicted because of following what their peers were doing. The desire to try out new things and be a part of a social network contributes to nyaope use (Marks, Gumede & Shelly 2017). Peer pressure has been identified as one of the most powerful predictors of youth behaviour, thus influencing drug experimentation. In a study by the Gauteng Department of Community Safety as reported by (Charlton et al. 2019), participants reported using nyaope to impress their friends to avoid being stigmatised. However, they became habitual users and were eventually addicted (Charlton et al. 2019). The participants felt that they would not be part of the social network if they did not use nyaope. Peer pressure is the strongest predictor of youth behaviour, having a huge impact on young people’s drug experimentation during adolescence (Dintwe 2017). One of the reasons for this is that young people want to be admired and rewarded by their peers.

Dagga is a gateway and reverse gateway for hard drugs (Mokwena 2019). This implies that it leads to the use and abuse of other more dangerous drugs. In addition, smoking dagga increases the likelihood of being misled by friends to smoke nyaope without one’s knowledge. The participants could not have been misled into smoking nyaope by their friends if they were not smoking dagga. This is corroborated by Marks and colleagues (2017) in their study where some nyaope users were uninformed of what drug was being used. They assumed they were smoking dagga whilst they were smoking nyaope. They became aware when they started to experience withdrawals after stopping their day-to-day use of the drug. This happened after a period of time sufficient to develop a physical dependence. Smoking nyaope turned into a way to cope with the pain and discomfort of the withdrawals, rather than enjoyment.

Overweight persons are more likely than their slimmer age group to face teasing, discrimination and tense relationships with family (Madowitz et al. 2012). Being overweight is associated with shame, guilt and social anxiety (Lavallee et al. 2021). Participants did not wish to experience discrimination. As a result, they found themselves applying mechanisms that would enable them to lose weight and keep their desired body image. Harriger and Thompson (2012) concurred with Lavallee and colleagues by reporting that body dissatisfaction is linked to low self-esteem and depression. A desire to lose weight has contributed to the usage of nyaope amongst the participants. Health and wellness centres must be established across communities, funded by the Department of Health to enable the community to access professional services whenever they need aid in losing weight, instead of using drugs or other unscientific methods. The wellness infrastructure should be of good quality to attract people to access the services.

Trauma contributes to the development of mental health difficulties and increases the likelihood of drug use (Arpa 2017); people frequently use substances to cope with negative experiences and emotions because these drugs provide temporary pleasure and enjoyment (Mokwena et al. 2021). According to some research, women are more prone than males to use substances to cope with mental health difficulties including depression, anxiety and post-traumatic stress disorder, which are frequently the outcome of trauma, abuse and violence (Pinkham & Malinowska-Sempruch 2008). Women who take drugs are said to have a high percentage of post-traumatic stress disorder and may have also experienced childhood problems such physical neglect, abuse or sexual abuse (UNODC 2018). Mental health experience can also play a role in rising women’s drug use and constraining their ability to distinguish and navigate risky circumstances (Stoicescu et al. 2018).

The participants started using nyaope to counteract other drugs. The participants began using nyaope to address restlessness, which was prompted by the use of the stimulant drug. It was never their intention to be regular users, but because of the addictiveness of nyaope, the participants found themselves dependent on nyaope. According to Masombuka (2013), cocaine temporarily reduces the demand for food and sleep, hence users have trouble sleeping after using it. Opioids, on the other hand, have the reverse effect: drowsiness and exhaustion are prominent opioid side effects (Angarita et al. 2016).

The study further affirms that a desire to feel a more significant ‘high’ and ‘pleasure’ influenced transitioning from smoking nyaope to injecting nyaope. Guise et al. (2017) land Baluku and Wamala (2021) corroborated this report that many studies described the search for a greater ‘high’ or ‘rush’, potentially available through injecting, as the primary reason linked to injection initiation. An increased high associated with drug injecting was described as developing from engagement in social networks that include people who already inject drugs, where the high is witnessed or encouraged and curiosity generated. In addition, the injecting of heroin results in near-rapid analgesic and ecstatic effects and the effect of heroin generally last for longer duration (Masombuka 2013).

Women who inject drugs are more likely to be in an intimate relationship with a partner who injects drugs. Their relationships with intimate male partners who also use drugs are complex and lively (Azim et al. 2015). In addition, they are more likely to have their first drug injecting experience with an intimate partner (El-Bassel & Strathdee 2015; Larney et al. 2015). This makes the women susceptible to the risk of blood-borne infection transmission during injection initiation, as they are more likely to be introduced by an intimate male partner, share drug injecting equipment and be injected after their initiator (Meyers et al. 2018). Refusing to use a partner’s used injecting equipment puts women at danger of IPV, which raises their HIV infection risk (GCWA 2011).

Social connections and networks were reported as fundamental to drug injecting initiation. Studies suggested that people who already inject may encourage injection initiation and excite none injectors about the benefits of drug injection, which is linked to pleasure or cost-efficiency (Guise et al. 2017). This advocacy is then linked to providing injection initiation assistance, including administering an individual’s first injection. This encouragement by people who already inject could extend to peer pressure and more direct coercion (Guise et al. 2017). It is common for experienced women who inject drugs to inspire their non-injecting friends to adjust the way of their administration to injection by explaining the profits of injecting drug use, such as the better, faster high than the cocktail route of administration (Tuchman 2015). This study’s findings are consistent with earlier research (Guise et al. 2017; Tuchman 2015), which found that being exposed to injection networks and having an intimate partner who is also an injector may influence injection initiation amongst women. Blood-borne infections are efficiently spread through the use of contaminated injection equipment and can spread quickly within PWID networks, and the availability of injecting equipment can dramatically reduce HIV transmission amongst women who inject drugs (Scheibe et al. 2017:2). It is critical that such networks have assistance with injecting equipment in order to avoid equipment sharing, which will contribute to an increase in the rate of blood-borne diseases.

Furthermore, the findings of this study highlight that, women who inject drugs are not always inactive in the transition process. Often, they had an active role in the transition. Some participants reported that injecting for the first time was their idea induced by curiosity. The findings of this study contradict the majority of the literature (El-Bassel & Strathdee 2016; GCWA 2011; Tuchman 2015; UNODC 2018) which states the influence of a significant other in initiating injecting nyaope amongst women. It is not always men who influence women to inject. For example, a female participant influenced her intimate male partner to inject nyaope whilst other participants injected because of curiosity after seeing other people injecting.

Injecting drugs is said to be a simple and effective way to consume nyaope. The injecting of nyaope produces near-rapid analgesic and ecstatic effects. When nyaope is injected, it lasts for a long period of time than when it is smoked (Masombuka 2013). Nyaope costs between R25–R35 per ‘fix’. However, it is an increasing cost. Even in the short term, it is high because of the strength of the addiction and the resultant frequent use (Mokwena & Morojele 2014). Nyaope injecting, on the other hand, is less expensive, prompting many users to switch from smoking to injecting, further increasing the chance of needle sharing and risk of overdose. The injecting practice must be continuously monitored and harm reduction programmes must be adopted to reduce blood-borne diseases amongst PWID. Whilst all methods of drug use have the potential for social and health consequences, injecting practice whether intravenous, subcutaneous or intramuscular is the technique of delivery that carries the greatest risk of infection, overdose and its sequelae (Baluku & Wamala 2019).

## Strengths and limitations

The strength of the study is, this research focused on women who are injecting nyaope. Women who inject nyaope are often overlooked and they were difficult to recruit to participate in the study, as they are hidden and scattered. The sample consists of 24 women participants. The study is the first to study exclusively women who inject nyaope as a population in SA.

The limitations of the study are as follows: the study included only participants who were accessing a service at selected COSUP sites. The research employed a qualitative research design; the findings cannot be generalised to all women who inject nyaope. Few studies were conducted on PWID within the South African context; UNODC conducted only one study on female nyaope users in SA in 2017. The researcher depended on international research for literature. Finally, the sample consisted of WWID regardless of whether they stay with family or are homeless, employed or unemployed.

## Recommendations

### Recommendation based on the findings of the research

- Dagga remains a gateway drug; an intense programme on the risk of using dagga should be implemented as part of the Department of Education curriculum.
- Mental health services should be easily accessible; the findings highlight that woman use drugs because of a lack of effective coping mechanisms, which mental health services can address.
- To prevent blood-borne infections among PWID, injecting practice must be regularly monitored and harm reduction initiatives must be implemented.

### Recommendation based on future research

- More studies on women who inject drugs should be conducted to understand better the South African drug injecting context, instead of understanding the South African drug injecting context using international research.
- A follow up quantitative study on factors contributing to the use of nyaope should be conducted.

### Recommendation based on policy and practice

- Programmes that educate drug injectors on safe injecting practices should be funded and upscaled
- People who inject drugs are a highly vulnerable group with high statistics of morbidity and premature mortality, and they should embrace harm reduction services that can offer support financially.

## Conclusion

The use of nyaope amongst women is a reality, which researchers and policymakers need to appreciate and study. The continual neglect of research focusing on WWUD has negative implications on policy and practice. More research on the factors contributing to smoking and the transition to injecting would inform practice and policy. This research has described that intimate partner influence, peer pressure, misled by friends, weight loss, lack of effective coping mechanism and counteracting other drugs have been reported to influence women to smoke nyaope. In contrast, the need for intense high, intimate partner influence, influence by the social network, curiosity and cost-effectiveness contributed to the transition from smoking to injecting nyaope amongst women. The study’s findings may be applicable to other injectable substances such as methamphetamine, cocaine, ecstasy and ketamine.
